# *LOXL1-AS1* contributes to metastasis in sonic-hedgehog medulloblastoma by promoting cancer stem-like phenotypes

**DOI:** 10.1186/s13046-024-03057-0

**Published:** 2024-04-30

**Authors:** Anh Duy Do, Kuo-Sheng Wu, Shing-Shung Chu, Le Hien Giang, Yu-Ling Lin, Che-Chang Chang, Tai-Tong Wong, Chia-Ling Hsieh, Shian-Ying Sung

**Affiliations:** 1 International Ph.D. Program for Translational Science, College of Medical Science and Technology, Taipei Medical University, Taipei, 11031 Taiwan; 2 Department of Physiology, Pathophysiology and Immunology, Pham Ngoc Thach University of Medicine, Ho Chi Minh City, 700000 Vietnam; 3 Graduate Institute of Clinical Medicine, School of Medicine, College of Medicine, Taipei Medical University, Taipei, 11031 Taiwan; 4 Department of Biology and Genetics, Hai Phong University of Medicine and Pharmacy, Hai Phong, 180000 Vietnam; 5 Agricultural Biotechnology Research Center, Academia Sinica, Taipei, 11529 Taiwan; 6 The Ph.D. Program for Translational Medicine, College of Medical Science and Technology, Taipei Medical University, Taipei, 11031 Taiwan; 7 Neuroscience Research Center, Taipei Medical University Hospital, Taipei, 11031 Taiwan; 8 Pediatric Brain Tumor Program, Taipei Cancer Center, Taipei Medical University, Taipei, 11031 Taiwan; 9 Division of Pediatric Neurosurgery, Department of Neurosurgery, Taipei Neuroscience Institute, Taipei Medical University Hospital, Taipei Medical University, Taipei, 11031 Taiwan; 10 Institute for Drug Evaluation Platform, Development Center for Biotechnology, Taipei, 11571 Taiwan; 11 TMU Research Center of Cancer Translational Medicine, Taipei Medical University, Taipei, 11031 Taiwan

**Keywords:** Medulloblastoma, Long non-coding RNA, Cancer stemness, Metastasis, LOXL1-AS1, TGF-β2

## Abstract

**Background:**

Medulloblastomas (MBs) are one of the most common malignant brain tumor types in children. MB prognosis, despite improvement in recent years, still depends on clinical and biological risk factors. Metastasis is the leading cause of MB-related deaths, which highlights an unmet need for risk stratification and targeted therapy to improve clinical outcomes. Among the four molecular subgroups, sonic-hedgehog (SHH)-MB harbors clinical and genetic heterogeneity with a subset of high-risk cases. Recently, long non-coding (lnc)RNAs were implied to contribute to cancer malignant progression, but their role in MB remains unclear. This study aimed to identify pro-malignant lncRNAs that have prognostic and therapeutic significance in SHH-MB.

**Methods:**

The Daoy SHH-MB cell line was engineered for ectopic expression of *MYCN*, a genetic signature of SHH-MB. MYCN-associated lncRNA genes were identified using RNA-sequencing data and were validated in SHH-MB cell lines, MB tissue samples, and patient cohort datasets. SHH-MB cells with genetic manipulation of the candidate lncRNA were evaluated for metastatic phenotypes in vitro, including cell migration, invasion, sphere formation, and expressions of stemness markers. An orthotopic xenograft mouse model was used to evaluate metastasis occurrence and survival. Finally, bioinformatic screening and in vitro assays were performed to explore downstream mechanisms.

**Results:**

Elevated lncRNA *LOXL1-AS1* expression was identified in MYCN-expressing Daoy cells and *MYCN*-amplified SHH-MB tumors, and was significantly associated with lower survival in SHH-MB patients. Functionally, *LOXL1-AS1* promoted SHH-MB cell migration and cancer stemness in vitro. In mice, MYCN-expressing Daoy cells exhibited a high metastatic rate and adverse effects on survival, both of which were suppressed under *LOLX1-AS1* perturbation. Integrative bioinformatic analyses revealed associations of *LOXL1-AS1* with processes of cancer stemness, cell differentiation, and the epithelial-mesenchymal transition. *LOXL1-AS1* positively regulated the expression of transforming growth factor (TGF)-β2. Knockdown of TGF-β2 in SHH-MB cells significantly abrogated their *LOXL1-AS1*-mediated prometastatic functions.

**Conclusions:**

This study proved the functional significance of *LOXL1-AS1* in SHH-MB metastasis by its promotion of TGF-β2-mediated cancer stem-like phenotypes, providing both prognostic and therapeutic potentials for targeting SHH-MB metastasis.

**Supplementary Information:**

The online version contains supplementary material available at 10.1186/s13046-024-03057-0.

## Background

Medulloblastomas (MBs) are one of the most common malignant brain tumor types in children [1]. Although the 5-year overall survival from MB has been improved to 70% ~ 80% [2–4], approximately one-third of patients surviving the primary tumor still suffer from recurrence, mostly presented with metastasis [5]. Metastatic disease in MB comes with the highest fatality, is hard to predict, and lacks a standard regimen [6]. Risk-adapted treatment approaches are currently optimized to maintain the survival rate but also to spare or limit the irradiation dose to avoid long-term neurocognitive effects [3, 4, 7]. The need for molecular definition of high-risk cases and targeted therapy options remains unmet for MB, mainly due to our insufficient understanding of molecular factors that drive MB malignancy.

MBs are classified into four molecular subgroups, namely wingless (WNT)-activated, sonic-hedgehog (SHH)-activated, Group 3, and Group 4, which are considered different entities due to their distinct genetic and clinical characteristics [8]. Accounting for ∼30% of all MB cases, SHH-MB has an intermediate to good prognosis in children and a well-characterized molecular profile [3, 4, 8, 9]. However, this is also the most heterogeneous subgroup with significant biological variations and unfavorable outcomes in a subset of cases [7, 9–11]. This phenomenon can be linked to genetic alterations that support malignant phenotypes of tumor cells. Among such drivers, the *MYCN* proto-oncogene is a target of SHH signaling pathway and also a signature gene of SHH-MB [12, 13]. Both *MYCN* amplification and MYCN overexpression are frequently observed in SHH-MB compared to other subgroups [8, 12, 14, 15]. *MYCN*-amplified tumors are relatively rare, but have poor prognosis and high resistance to smoothened (SMO) inhibitors [11, 14, 16–18]. MYCN ectopic expression in neural/neuroepithelial stem cells can generate highly proliferative and metastatic brain tumors in vivo, whose transcriptomes resemble SHH-MB with enhanced stemness signatures [19–21]. Nevertheless, MYCN remains a very hard-to-drug target due to its pleiotropic roles in both cancer and fetal cerebellar development [15].

Tumor aggressiveness driven by MYCN can be acquired via dysregulation of MYCN-dependent genes, including long non-coding (lnc)RNAs, a type of non-coding RNAs. LncRNAs were recently characterized as specific regulators of diverse cancer behaviors, although our understanding about their roles in MB is just emerging [22, 23]. Interestingly, SHH-MB has a unique landscape of non-coding transcripts with potential relevance in tumor biology [24]. Identification of novel lncRNAs that contribute to SHH-MB metastasis can support practical strategies for risk stratification, regimen optimization, and targeted therapy development to further improve clinical outcomes.

In this study, we applied integrative screening approaches to identify lncRNA genes associated with MYCN expression in SHH-MB, among which lysyl oxidase-like 1-antisense 1 (*LOXL1-AS1*) appeared as a potential candidate. We sought to determine this lncRNA’s clinical relevance, its functions related to metastatic phenotypes, and its putative molecular effectors in SHH-MB.

## Methods

### Clinical samples

This study included 70 MB cases from 1989 to 2019 at Taipei Veterans General Hospital (Taipei VGH) and Taipei Medical University Hospital (TMUH). All patients gave written informed consent to provide tissues and clinical data for research purposes. The collection and use of human specimens complied with the *Declaration of Helsinki* and followed a protocol approved by the Institutional Review Board of Taipei Medical University (IRB approval nos. 201701441A3 and N202202069). MB tissues obtained from the Taipei Medical University Joint Biobank (TMU-JBB) were separately processed to make a frozen tumor tissue repository and paraffin-embedded blocks for DNA/RNA profiling extraction and histological study, respectively.

### Bioinformatic analysis

DNA/RNA profiling and clinical data of our MB cohort (*n* = 70) were described in a previous study [25]. For validation, an independent and non-overlapping MB cohort (*n* = 264) as part of a comprehensive dataset of pediatric tumors (OpenPedCan vers. 12) was retrieved from the Pediatric cBioPortal platform (https://pedcbioportal.org). Other MB datasets used in this study were accessed for analysis through the R2 public genomic archive platform (https://r2.amc.nl). Gene expression as normalized RNA-Seq read counts were retrieved for analyses. Differentially expressed gene (DEG) analysis and gene set enrichment analysis (GSEA) were performed using suitable packages in R (vers. 3.6.1).

### Cell cultures

Two human SHH-MB cell lines, Daoy and ONS76, were originally obtained from American Type Culture Collection (ATCC, Manassas, VA, USA). Daoy cells were cultured in Eagle’s minimum essential medium (Cytiva, Marlborough, MA, USA) supplemented with 10% fetal bovine serum (FBS) (Cytiva) and 1% penicillin-streptomycin (PS) (Cytiva). ONS76 cells were cultured in RPMI 1640 medium supplemented with 10% FBS and 1% PS. HEK293FT cells obtained from Invitrogen (ThermoFisher Scientific, Waltham, MA, USA) were cultured in high-glucose Dulbecco’s modified Eagle medium (DMEM) (Cytiva) supplemented with 10% FBS, 1% PS, and 1% non-essential amino acids (Cytiva). Cells were incubated at 37 °C in 5% CO_2_ and were routinely passaged upon 90% confluence.

### Gene manipulation

For MYCN overexpression, complementary DNA (cDNA) of human *MYCN* (clone ID 3627330, Horizon Discovery, Cambridge, UK) was cloned into the pLenti4-Flag-CPO-v2 expression vector through the service of the National RNAi Core Facility (Academia Sinica, Taipei, Taiwan). For gene knockdown, small-hairpin (sh)RNAs targeting *LOXL1-AS1* (shLOXL1-AS1) in the LV2N-U6/Puro vector were purchased from GenePharma (Shanghai, China), and shRNAs targeting *TGFB2* (shTGFB2) in the pLKO.1-Puro vector were purchased from the National RNAi Core Facility (Academia Sinica). Detailed information about shRNA targeting sequences is given in Supplementary Table S1. The cDNA- or shRNA-containing lentiviruses were produced by transfection of HEK293FT cells using the Maestrofectin transfection reagent (Omics Bio, Taipei, Taiwan) according to the manufacturer’s protocol. Lentiviral transduction was performed on MB cells followed by selection with 250 μg/mL zeocin or 2.5 μg/mL puromycin to establish stable lines. For *LOXL1-AS1* overexpression, the full-length cDNA of the *LOXL1-AS1* transcript (2501 bp, NCBI accession no. NR_040066.1) in the pcDNA3.1 expression vector and the empty pcDNA3.1 vector as negative control were purchased from GenePharma, then subjected to direct transfection into SHH-MB cells using the Maestrofectin transfection reagent, followed by selection with 200 μg/mL G418 to establish a stable cell line.

### Sphere-forming culture and sphere-formation assay

Monolayer cells were first detached with Accutase (BD Biosciences, Franklin Lakes, NJ, USA) until complete separation into single cells. Then, cells were washed three times with 1× phosphate-buffered saline (PBS) and seeded into ultra-low-attachment T175 flasks in a defined culture serum-free medium composed of DMEM/F12 (Cytiva) supplemented with 2% B-27 (ThermoFisher Scientific), 20 ng/mL of recombinant human basic fibroblast growth factor (bFGF; R&D Systems, Minneapolis, MN, USA), 20 ng/mL of recombinant human epidermal growth factor (hEGF; R&D Systems), and 1% PS [26]. A very low seeding density of 2000 cells/mL was applied to minimize cell aggregation. Fresh culture medium was added every 3 ~ 4 days for 2 weeks. Then, the entire culture of each sample was subjected to sphere counting, RNA extraction, or protein extraction. To evaluate sphere numbers and sizes, cells were gently re-distributed into a 96-well plate with a 3-mL plastic pipette for microscopic images, in which every spherical structure with a diameter of ≥100 μm was counted as a sphere using Fiji/ImageJ software. Sphere-formation efficiency was calculated as the number of spheres per 1000 cells seeded.

### Cell migration and invasion assays

For the wound-healing assay, two- or three-well culture inserts (Ibidi, Gräfelfing, Germany) were placed in a culture dish, with 2.5 × 10^4^ cells in 100 μL culture medium seeded into each well. After overnight incubation, the inserts were removed to create a gap. Cells were gently washed with 1× PBS and replenished with fresh culture medium for continued incubation. Microscopic images of the gap were captured at indicated time points. The gap area was quantified using Fiji/ImageJ software. For the transwell migration and invasion assays, hanging culture inserts with an 8-μm-pore membrane (Merck Millipore, Burlington, MA, USA) were placed in a 24-well plate. Additionally, the membrane was pre-coated with 50 μL of 1 mg/mL Matrigel (Life Sciences, Corning, NY, USA) in the invasion assay. In total, 5 × 10^4^ cells in 200 μL serum-free medium were seeded into the insert (upper chamber) and 800 μL of culture medium with 1% FBS was added to the well (lower chamber). After incubation for 3 h for migration or 12 h for invasion, cells inside the insert were gently removed, and cells on the outside surface were fixed and stained with 0.5% crystal violet. Five random fields at 20× magnification were captured for automated cell counting using Fiji/ImageJ software. Alternatively, crystal violet from stained cells was dissolved in 100 μL of Sorenson’s solution and then quantified by measuring the absorbance at 570 nm.

### RNA-sequencing (RNA-Seq) and real-time reverse-transcription quantitative polymerase chain reaction (RT-qPCR) analysis

Total cellular RNA was extracted with NC RNA extraction reagent (EBL Biotechnology, Taipei, Taiwan) following the manufacturer’s instructions. Cellular RNA samples were sent to the Taipei Medical University Core Facility Center for library preparation and sequencing. A PrimeScript RT reagent kit (Takara Bio, Shiga, Japan) was used for RNA reverse-transcription into cDNA. Then, a real-time RT-qPCR was performed using gene-specific primer pairs (listed in Supplementary Table S2), a QuantiNova SYBR Green PCR kit (Qiagen, Hilden, Germany), and LightCycler 96 (Roche, Basel, Switzerland), all according to the manufacturers’ instructions. The 2^-ΔΔCt^ method was applied to calculate gene expressions, with *HSPCB* being the housekeeping gene for normalization.

### Protein extraction and Western blot analysis

Whole-cell lysates were collected, determined of protein concentration, and blotted following a previously described protocol [27] using specific antibodies: anti-N-Myc (cat. no. D4B2Y #51705, Cell Signaling Technology, Danvers, MA, USA), anti-Sox2 (cat. no. GTX101507, GeneTex, Irvine, CA, USA), anti-Oct4 (cat. no. GTX101507, GeneTex), anti-Nanog (D73G4, cat. no. #4903, Cell Signaling Technology), anti-Bmi1 (D20B7, cat. no. #6964, Cell Signaling Technology), anti-TGF-β2 (cat. no. MAB612, R&D Systems), and anti-β-actin (cat. no. GTX109639, GeneTex). All first antibodies were probed at a 1:1000 dilution overnight at 4 °C, while second antibodies, including anti-mouse (cat. no. 111-035-146, Jackson ImmunoResearch, West Grove, PA, USA) and anti-rabbit (cat. no. 111-035-144, Jackson ImmunoResearch), were probed at a 1:10^4^ dilution for 1 h at room temperature. Blots were visualized using a chemiluminescent detection kit (T-Pro Biotechnology, New Taipei City, Taiwan) and Amersham Imager 600 (Cytiva Life Sciences). Fiji/ImageJ software was used to quantify bands with all values normalized to the β-actin signal.

### In situ hybridization and immunohistochemical (IHC) staining

In situ hybridization was performed using an RNAScope 2.5 HD Assay-RED kit (Advanced Cell Diagnostics, Newark, CA, USA) following the manufacturer’s protocol. Gene-specific probes for human *LOXL1-AS1* with human peptidyl-prolyl cis-trans isomerase B (*PPIB*) as a positive control and bacterial *dapB* as a negative control were designed by the manufacturer. RNA expression was determined by automated detection of chromogenic dots using the Color Deconvolution and Weka Classifiers plug-ins and was normalized to the positive control RNA signal. IHC staining was performed with an anti-Mycn antibody (cat. no. #51705, Cell Signaling Technology) and Novolink Polymer Detection System kit (Leica Biosystems, Richmond, IL, USA) according to the manufacturer’s instructions. Protein expression was determined by quantification of the chromogenic signal intensity. All images were analyzed with Fiji/ImageJ software.

### Animal studies

This study was approved by the Taipei Medical University (TMU) Ethics Committee for the use of a xenograft brain tumor model (LAC-2021-0475). Luciferase-tagged Daoy or Daoy-MYCN cells were prior checked for stable luciferase activity and the absence of mycoplasma infection, and then washed and suspended in 1× PBS and Matrigel (Life Sciences) at a ratio of 2:1. In total, 4 × 10^5^ cells in 10 μL were injected into the left cerebral hemisphere (2.5 mm lateral, 0.14 mm posterior to the bregma, and 3 mm in depth) of 6 ~ 8-week-old NOD/SCID mice (National Laboratory Animal Center, Taipei, Taiwan). Bioluminescence imaging (BLI) was performed at 3 and 7 days post-inoculation (dpi) to confirm the presence of xenograft cells and then weekly until the endpoint at day 63 (for Daoy vs. Daoy-MYCN) or day 56 (for Daoy-MYCN shNC vs. shLOXL1-AS1). The brain and spinal cord were obtained post-mortem for a histological study. All animal care, monitoring, and experiments followed guidelines of the TMU Laboratory Animal Center where the animal study was conducted.

## Statistical analysis

Data are presented as the mean ± standard deviation (SD) with the difference between two groups determined by a two-tailed Student’s *t*-test unless otherwise specified in the figure legends. Correlations between two variables were assessed using a simple linear regression and Pearson’s correlation coefficients. Survival analyses were carried out using a Kaplan-Meier curve and the Gehan-Breslow-Wilcoxon test. The cut-off values of gene expression for samples grouping was determined using the *maxstat* and *survminer* packages (*surv_cutpoint* function, *minprop* = 0.25) in R software. All statistical analyses were performed with GraphPad Prism vers. 9 (GraphPad, San Diego, CA, USA) and Microsoft Office Excel vers. 16 (Microsoft, Redmond, WA, USA). *p* < 0.05 (*) was considered statistically significant.

## Results

### Establishment of a MYCN-driven SHH-MB model with a high rate of metastasis in vivo

**Fig. 1:**
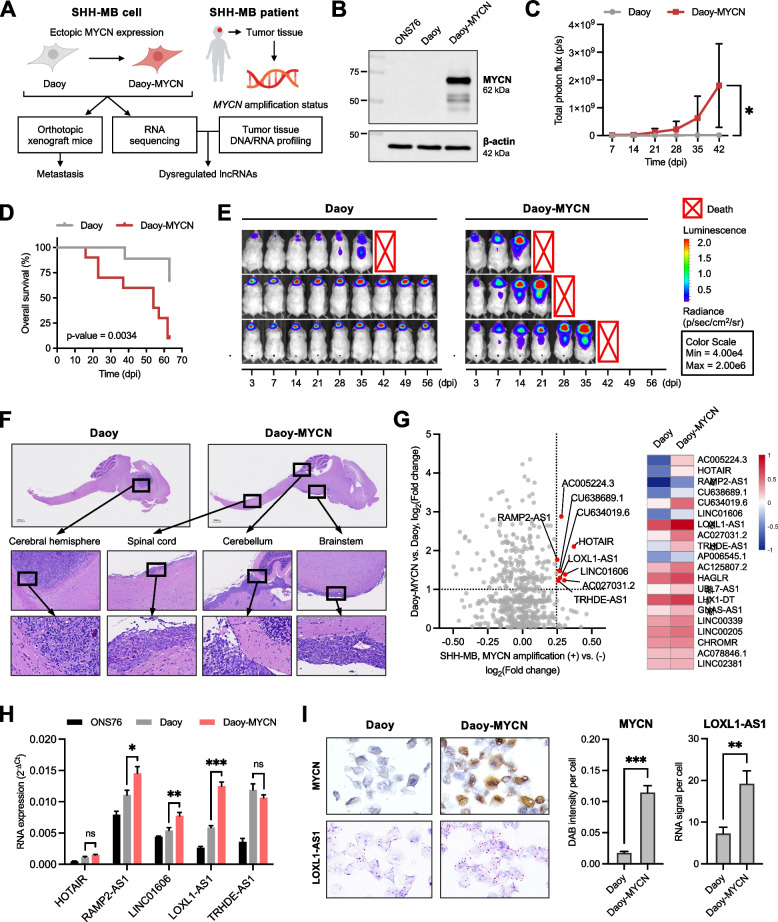
Identification of dysregulated long non-coding (lnc)RNA targets in highly malignant MYCN-expressing sonic-hedgehog medulloblastoma (SHH-MB) cells and clinical samples. **A** Schematic outline of the experiment design. **B** Western blot analysis of MYCN expression in ONS76, Daoy, and Daoy-MYCN cells. **C-E** NOD/SCID mice were randomly divided into two groups for orthotopic inoculation with Daoy or Daoy-MYCN cells. Data are presented as (C) total photon flux measurements, (D) overall survival analysis, and (E) BLI of representative mice in each group from 3 to 56 dpi. **F** H&E staining images of the brain and spinal cord of a representative mouse from each group. **G** Scatterplot (left) showing all non-coding genes found in SHH-MB clinical samples (*MYCN* amplification positive versus negative, x-axis) and in cell line samples (Daoy-MYCN vs. Daoy, y-axis). Genes differentially expressed in both *MYCN*-amplified tumors and Daoy-MYCN cells are labeled with gene names and marked as red dots. Heatmap (right) of the top differentially expressed genes in *MYCN*-amplified SHH-MB tumors with their expression levels in Daoy and Daoy-MYCN cells. **H** RT-qPCR validation of five annotated lncRNA genes in ONS76, Daoy, and Daoy-MYCN cells. **I** Immunohistochemical staining of MYCN protein and in situ hybridization of *LOXL1-AS1* RNA in Daoy and Daoy-MYCN cells (left images) and quantitative signals (right bar charts). Quantitative data are presented as mean ± SD of replicates from representative of three independent experiments. BLI, bioluminescence imaging; dpi, days post-inoculation. * *p* < 0.05, ** *p* < 0.01, *** *p* < 0.001

To target SHH-MB metastasis, we first aimed to develop a suitable model in xenograft mice. Previous studies demonstrated de novo SHH-MB formation in mice which had been transplanted with neural stem cells aberrantly expressing MYCN [19–21]. MYCN withdrawal, on the other hand, abated cancer stemness properties of tumor-propagating cells in a MYCN-dependent MB mouse model [28]. We hypothesized that in MB cells with a low MYCN level, MYCN overexpression could promote their malignant features to drive metastasis in vivo. Compared to other human MB cell lines, Daoy cells have a gene expression profile closely resembling SHH-MB and can recapitulate the cancer stemness of human brain tumor-initiating cells [29]. Therefore, we ectopically expressed MYCN in Daoy cells (Daoy-MYCN) and then inoculated these cells into mouse brains to observe tumor formation and metastasis (Fig. 1A-B). Compared to mice injected with Daoy, those injected with Daoy-MYCN exhibited early-onset metastasis, a striking increase in the tumor burden, and a lower survival rate (Fig. 1C-D). BLI signals were detected in the head region indicating intracranial tumors and in the body region indicating spinal cord metastases (Fig. 1E). Histological studies confirmed the presence of xenograft tumors at inoculation site in both groups, and particularly at other sites of the central nervous system, including the cerebellum, brainstem, and spinal cord, in Daoy-MYCN group (Fig. 1F). Therefore, MYCN overexpression produced a highly aggressive phenotype of Daoy cells observed in xenografted mice.

### Screening for dysregulated lncRNAs in SHH-MB cells identifies *LOXL1-AS1* as a potential MYCN-associated gene

To explore potential lncRNAs in malignant SHH-MB, we performed RNA-Seq to achieve the first record of MYCN-dependent lncRNAs in Daoy-MYCN cells. To ensure clinical relevance, we used DNA/RNA profiling data from 24 SHH-MB patients and grouped those harboring *MYCN* amplification (*n* = 4) to compare against the remaining cases, resulting in differentially expressed lncRNAs in these cases. Then, Daoy cell RNA-Seq data were integrated to further screen out the top nine genes with the highest expression levels in both *MYCN*-amplified tumors and MYCN-expressing cells (Fig. 1G). Five of these nine candidates have been fully annotated and were subjected to validation. Expression of the gene *LOXL1-AS1* was significantly elevated in Daoy-MYCN compared to Daoy in RT-qPCR (Fig. 1H), which was further confirmed by in situ hybridization (Fig. 1I). Explorative in silico genomic screening for predicted transcription factor-binding sites detected enrichment of a MYCN-binding motif upstream of the *LOXL1-AS1* transcription start site (Supplementary Fig. S1), suggesting a possible interaction via promoter region binding. Altogether, our integrative screening identified *LOXL1-AS1* as a potential MYCN-associated lncRNA in SHH-MB.

### *LOXL1-AS1* is highly expressed in MYCN-positive tissues and was correlated with poor outcomes in the SHH-MB subgroup

**Fig. 2:**
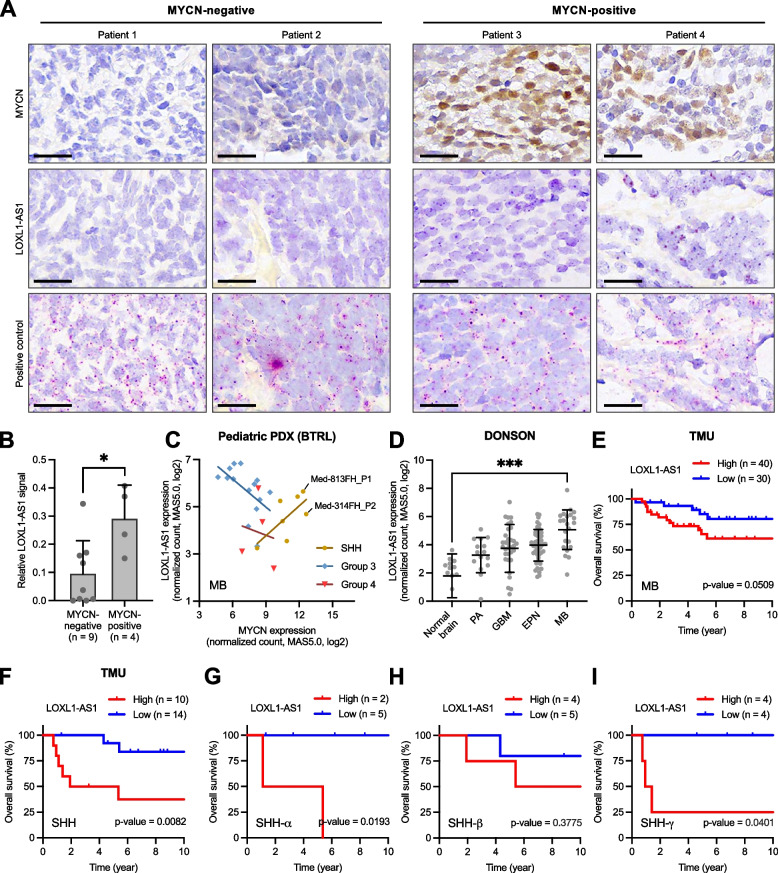
Tissue expression of *LOXL1-AS1* and its association with survival in sonic-hedgehog medulloblastoma (SHH-MB) cohorts. **A-B** Immunohistochemical staining of MYCN protein and in situ hybridization of *LOXL1-AS1* and positive control RNA in 13 SHH-MB samples divided into two groups based on the MYCN signal (positive or negative), including (A) representative staining images and (B) quantitative *LOXL1-AS1* signals. Scale bars, 20 μm. **C** Correlation between *MYCN* and *LOXL1-AS1* in MB primary cell lines (Brain Tumor Resource Lab). Two SHH cell lines with *MYCN* amplifications are specifically indicated. Simple linear regression analysis: SHH, *n* = 7, *R* = 0.687, *p* = 0.0883; Group 3, *n* = 14, *R* = -0.531, *p* = 0.0509; Group 4, *n* = 4, *R* = -0.144, *p* = 0.8557. **D** *LOXL1-AS1* expression in the normal brain and in different types of brain tumors in a public dataset (Donson, R2 platform). PA, pilocytic astrocytoma; GBM, glioblastoma; EPN, ependymoma; MB, medulloblastoma. **E-I** Survival analyses of *LOXL1-AS1* expression in our cohort (TMU), including (E) all MB cases, (F) the SHH subgroup of MB, and (G-I) three subtypes of SHH-MB. * *p* < 0.05, ** *p* < 0.01, *** *p* < 0.001

Next, we validated the relevance of *LOXL1-AS1* in SHH-MB clinical samples and cohorts. *LOXL1-AS1* RNA signals were found to be higher in MYCN-positive SHH-MB tissues than in MYCN-negative ones (Fig. 2A-B). A correlation between MYCN and *LOXL1-AS1* expression was found in primary cell lines of SHH-MB, despite statistical insignificance likely due to small sample size (Fig. 2C). Notably, two primary cell lines with *MYCN* amplification were also highly expressed with both MYCN and *LOXL1-AS1*. *LOXL1-AS1* expression was also higher in MB compared to the normal brain or other brain tumors (Figs. 2D, S2A). Survival analyses uncovered that high *LOXL1-AS1* expression was correlated with poor outcomes in MB regardless of the subgroup (Fig. 2E) and more significantly in the SHH subgroup (Figs. 2F, S2B). SHH-MB cases were further sub-categorized into three subtypes of SHH-α, -β, and -γ in children [9], where *LOXL1-AS1* expression was significantly associated with worse survival in SHH-α and SHH-γ patients (Fig. 2G-I). Collectively, *LOXL1-AS1* is a novel marker predictive of poor outcomes in SHH-MB.

### *LOXL1-AS1* enhances the stemness features and promotes the migratory and invasive abilities of SHH-MB cells in vitro

**Fig. 3:**
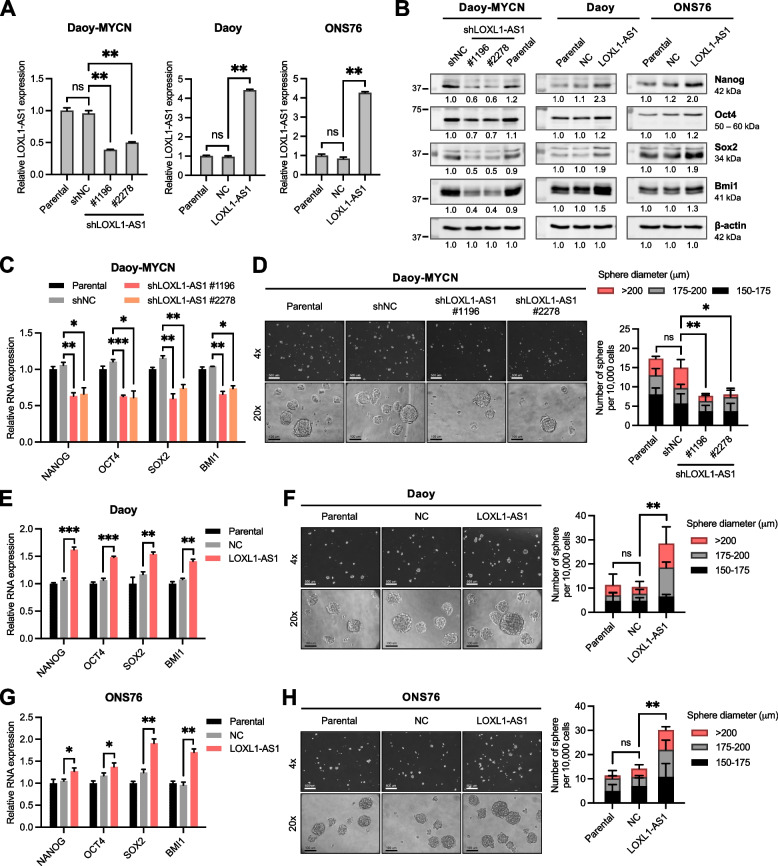
Effects of *LOXL1-AS1* on stemness phenotypes of sonic-hedgehog medulloblastoma cells. **A** RT-qPCR analysis of *LOXL1-AS1* expression in the Daoy-MYCN, Daoy, and ONS76 cell lines relative to the parental group. **B** Western blot analysis of four stemness markers in the Daoy-MYCN, Daoy, and ONS76 cell lines. Quantification values below each band are relative to the parental group. **C-H** RT-qPCR analysis of four stemness markers in the (C) Daoy-MYCN, (E) Daoy, and (G) ONS76 cell lines relative to the parental group. Sphere-formation assay of the (D) Daoy-MYCN, (F) Daoy, and (H) ONS76 cell lines, including microscopic images at different magnifications (left images) and the number and sizes of spheres of ≥150 μm in diameter (right bar chart). Scale bars, 500 μm in 4× and 100 μm in 20× magnification. Quantitative data are presented as mean ± SD of replicates from representative of three independent experiments. NC, negative control; ns, non-significant. * *p* < 0.05, ** *p* < 0.01, *** *p* < 0.001

**Fig. 4:**
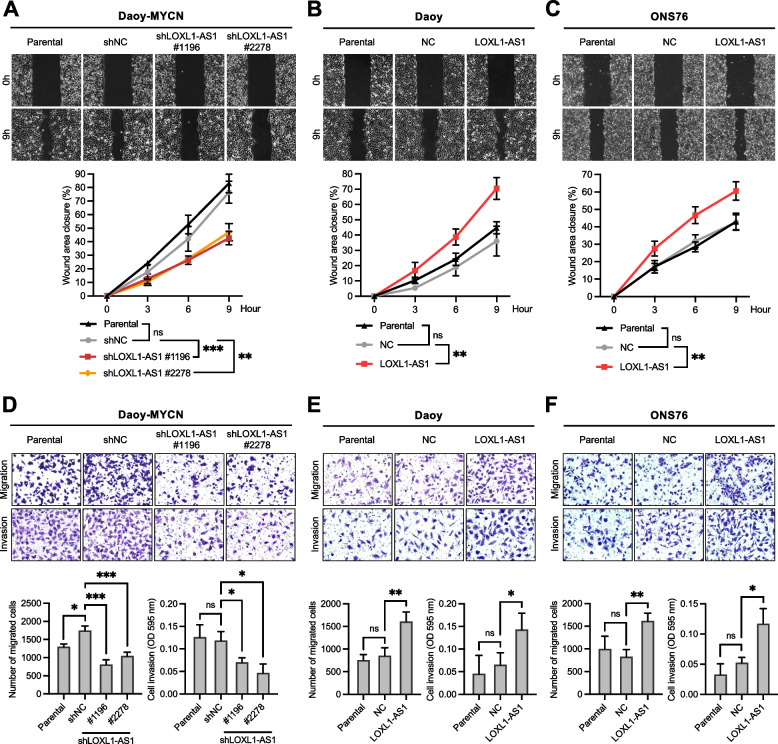
Effects of *LOXL1-AS1* on the migration and invasion capabilities of sonic-hedgehog medulloblastoma cells. **A-C** Wound-healing assay of the (A) Daoy-MYCN, (B) Daoy, and (C) ONS76 cell lines. **D-F** Transwell migration and invasion assays of the (D) Daoy-MYCN, (E) Daoy, and (F) ONS76 cell lines, including quantification of cell migration (left bar chart) and invasion (right bar chart). Quantitative data are presented as mean ± SD of replicates from representative of three independent experiments. NC, negative control; ns, non-significant. * *p* < 0.05, ** *p* < 0.01, *** *p* < 0.001

To investigate the effects of *LOXL1-AS1* in vitro, we silenced *LOXL1-AS1* expression in Daoy-MYCN cells and overexpressed *LOXL1-AS1* in Daoy and ONS76 cells (Fig. 3A). To evaluate stemness-related characteristics, we maintained SHH-MB cells in a defined sphere-forming condition widely used to enrich cancer stemness in vitro [26]. Results showed that the known stemness regulators, including Nanog, Oct4, Sox2, and Bmi1, decreased under *LOXL1-AS1* depletion and increased under ectopic *LOXL1-AS1* expression (Fig. 3B-C, E, G). The sphere-formation capacity, reflected by the sphere number and size, was significantly impaired in *LOXL1-AS1*-knockdown cells but was augmented in *LOXL1-AS1*-overexpressing cells (Fig. 3D, F, H). Furthermore, *LOXL1-AS1* silencing in Daoy-MYCN cells significantly reduced cell migratory phenotypes observed via wound-healing and transwell migration-invasion assays (Fig. 4A, D), while *LOXL1-AS1* overexpression in Daoy and ONS76 cells produced corresponding increases (Fig. 4B-C, E-F). Taken together, *LOXL1-AS1* facilitated metastatic features of SHH-MB cells, including expression of stemness factors, formation of tumor spheres, and the capabilities of cell migration and invasion in vitro.

### Perturbation of *LOXL1-AS1* in MYCN-expressing SHH-MB cells significantly delays early-onset metastasis and improves survival in vivo

**Fig. 5:**
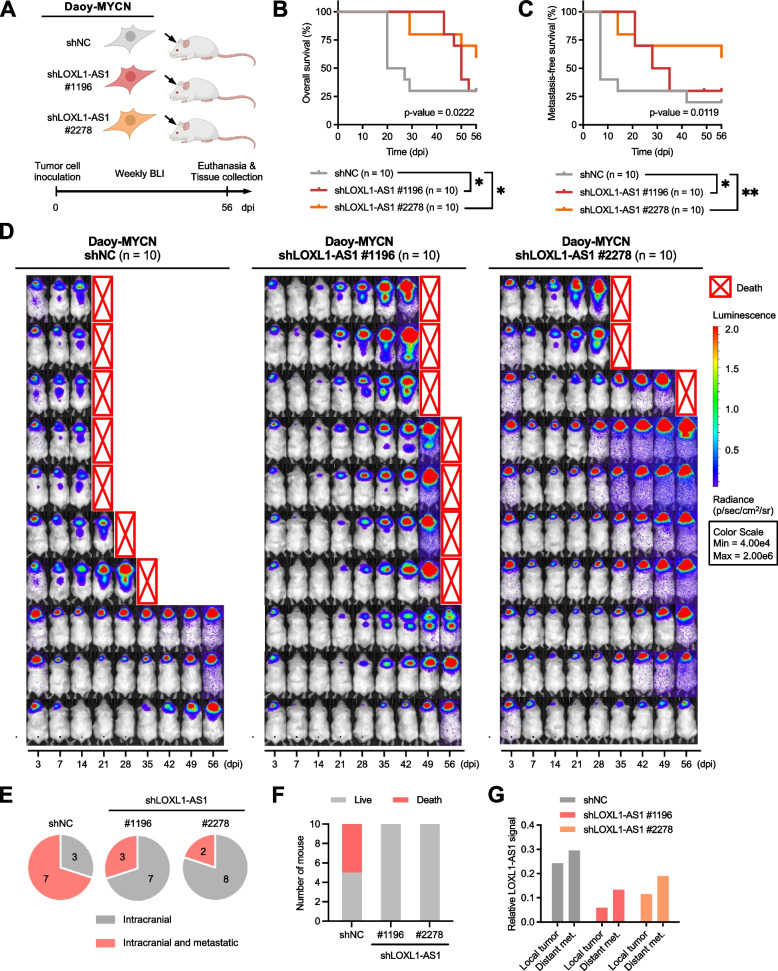
Effects of *LOXL1-AS1* perturbation on metastasis occurrence and survival rates in orthotopic xenograft mice. **A** Schematic outline of the animal study. NOD/SCID mice were randomly divided into three groups for inoculation with Daoy-MYCN -shNC, -shLOXL1-AS1 #1196, and -shLOXL1-AS1 #2278 cells. **B** Overall survival analysis of the three groups. **C** Metastasis-free survival analysis of the three groups. **D** BLI of all mice in each group from 3 to 56 dpi. **E-F** Data analysis at 21 dpi, including (E) the number of mice with only intracranial signals versus with both intracranial and metastatic signals and (F) the number of dead mice in each group. The presence and location of tumors were confirmed by a histological study of brains and spinal cords at the end-point. **G** *LOXL1-AS1* signal detected from in situ hybridization of the brain and spinal cord tissue of representative mice from each group. See also Supplementary Fig. S3. BLI, bioluminescence imaging; dpi, days post-inoculation. * *p* < 0.05, ** *p* < 0.01

To evaluate the effect of *LOXL1-AS1* depletion in vivo, the highly metastatic Daoy-MYCN cells with *LOXL1-AS1* knockdown were chosen for transplantation into mouse brains (Fig. 5A). *LOXL1-AS1*-knockdown resulted in significantly improved overall survival and metastasis-free survival (Fig. 5B-C). Although all mice eventually developed tumors, those injected with *LOXL1-AS1*-knockdown cells exhibited a notable delay in the occurrence of metastasis compared to those injected with knockdown-negative control cells (Fig. 5D). Seven of 10 mice in the control group experienced metastasis and five of them died rapidly within the first 3 weeks after inoculation (Fig. 5E). In contrast, mice in the *LOXL1-AS1*-knockdown groups had low metastasis rates and none died prior to 5 weeks of cell transplantation (Fig. 5F). These knockdown groups, however, experienced high primary tumor burdens later, most likely due to their prolonged survival time. Histological study of tissues collected at the end-point confirmed the presence of xenograft tumors at the site of inoculation and at other common sites of leptomeningeal spread (Supplementary Fig. S3). Interestingly, higher *LOXL1-AS1* signals were detected in metastatic lesions regardless of the mouse group (Fig. 5G). Our in vivo results suggested that *LOXL1-AS1* is a crucial factor not for primary tumor growth but for the development of leptomeningeal metastasis, which severely affects survival.

### RNA-Seq data analyses reveal *LOXL1-AS1* potentially regulating pathways and genes involved in cell migration and stemness-differentiation

**Fig. 6:**
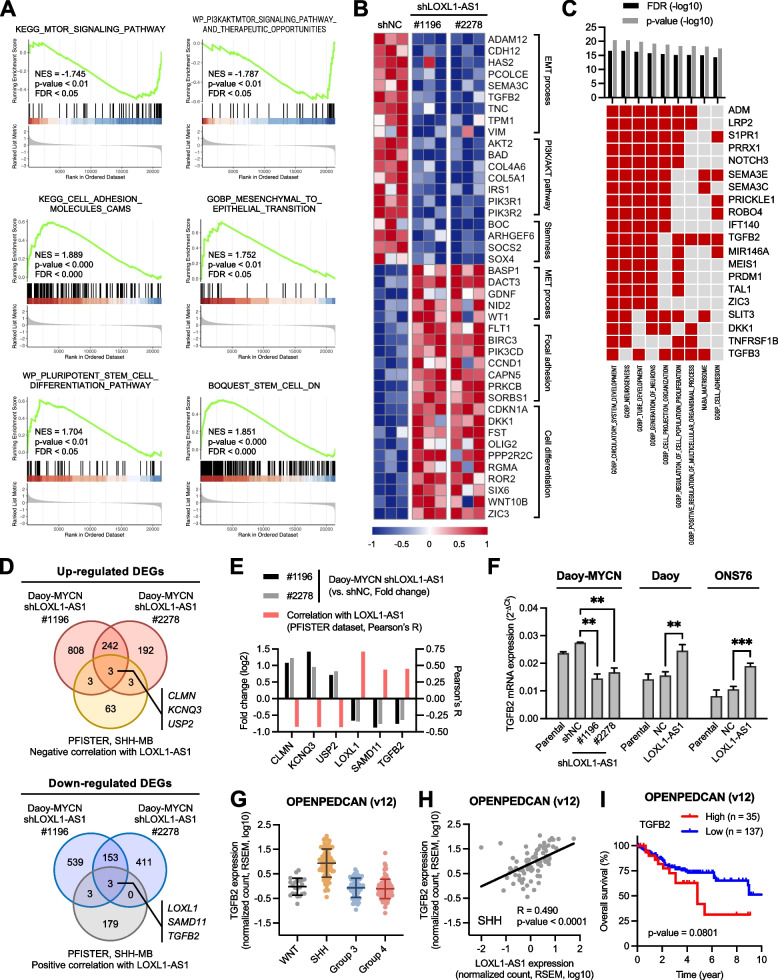
RNA-sequencing analyses of dysregulated pathways and putative effector genes of *LOXL1-AS1*. **A** Gene set enrichment analysis (GSEA) plots of Daoy-MYCN-shLOXL1-AS1 #1196 versus -shNC samples in six gene sets of interest. See also Supplementary Fig. S4A-B. **B** Heatmap of differentially expressed genes (DEGs) closely associated with pathways or processes of interest. **C** GSEA of the top DEGs found in both knockdown samples. Bar chart (top) shows the false discovery rate (FDR) and *p* value of the top nine enriched pathways. Matrix table (below) includes the top 20 enriched genes and their presence in each pathway (red, present; gray, absent). See also Supplementary Fig. S4C. **D** Venn diagrams of DEGs found in each knockdown sample overlapping with an additional set of genes that have strong correlations with *LOXL1-AS1* found in a public SHH-MB dataset (Pfister-223, R2 platform). Genes at the center overlap are specifically indicated. See also Supplementary Fig. S5A. **E** Bar chart of six candidate genes summarizing their differential expressions in SHH-MB cells (left y-axis) and correlations with *LOXL1-AS1* in SHH-MB patient dataset (right y-axis). **F** RT-qPCR validation of *TGFB2* in the Daoy-MYCN, Daoy, and ONS76 cell lines. Quantitative data are presented as mean ± SD of replicates from representative of three independent experiments. NC, negative control. ** *p* < 0.01, *** *p* < 0.001. **G-I** *TGFB2* expression in the OpenPedCan cohort including (G) expression levels across four molecular MB subgroups, (H) correlation with *LOXL1-AS1* in SHH-MB, and (I) survival analysis

LncRNAs can act via a wide range of mechanisms, many of which are linked to modulating gene expressions. To identify downstream targets responsible for the significant changes observed in Daoy-MYCN cells, we performed thorough RNA-Seq profiling of two knockdown samples (shLOXL1-AS1 #1196 and #2278) compared to the negative control (shNC). These RNA-Seq data were first subjected to unbiased GSEA screening (Supplementary Fig. S4A). The top up- and down-regulated gene sets with both significance and agreement between the two knockdown samples were identified and showed that *LOXL1-AS1* could be involved in the PI3K/AKT/mTOR pathway, epithelial-mesenchymal transition (EMT), and cell adhesion, differentiation, and stemness (Figs. 6A, S4B). Analysis of DEGs were explored to produce a curated list of important genes involved in each process (Fig. 6B). Next, we adopted a second approach in which totals of 401 DEGs in both knockdown samples were used as input for an investigative GSEA (Supplementary Fig. S4C). Results of the top enriched gene sets with the top overlapping genes revealed the involvement of neurogenesis, neuron generation, cell adhesion, and cell proliferation processes (Fig. 6C). These data suggested that the phenotypical changes observed upon *LOXL1-AS1* alteration in SHH-MB cells could result from dysregulation of pro-migratory and pro-stemness signaling pathways.

### TGF-β2 is a putative effector of *LOXL1-AS1* with prognostic value and prometastatic functions in SHH-MB

To identify the functional effector of *LOXL1-AS1*, we stringently narrowed down candidate genes which exhibited consistent associations with *LOXL1-AS1* in SHH-MB. A collection of DEGs found in Daoy-MYCN-shLOXL1-AS1 cells was intersected with another collection of genes correlated with *LOXL1-AS1* in SHH-MB tumors (Figs. 6D, S5A), resulting in six dysregulated candidate genes (Figs. 6E, S5B). RT-qPCR validation in SHH-MB cells further confirmed that *TGFB2*, encoding TGF-β2, had the most significant changes under *LOXL1-AS1* alterations (Figs. 6F, S5C). Importantly, *TGFB2* was among the genes participating in pathways discovered from our GSEA results (Fig. 6B, C). Among MB subgroups, *TGFB2* had the highest mRNA basal level and the strongest correlation with *LOXL1-AS1* in SHH-MB (Figs. 6G-H, S5D-E), as well as an association with unfavorable outcomes (Figs. 6I, S5F).

**Fig. 7:**
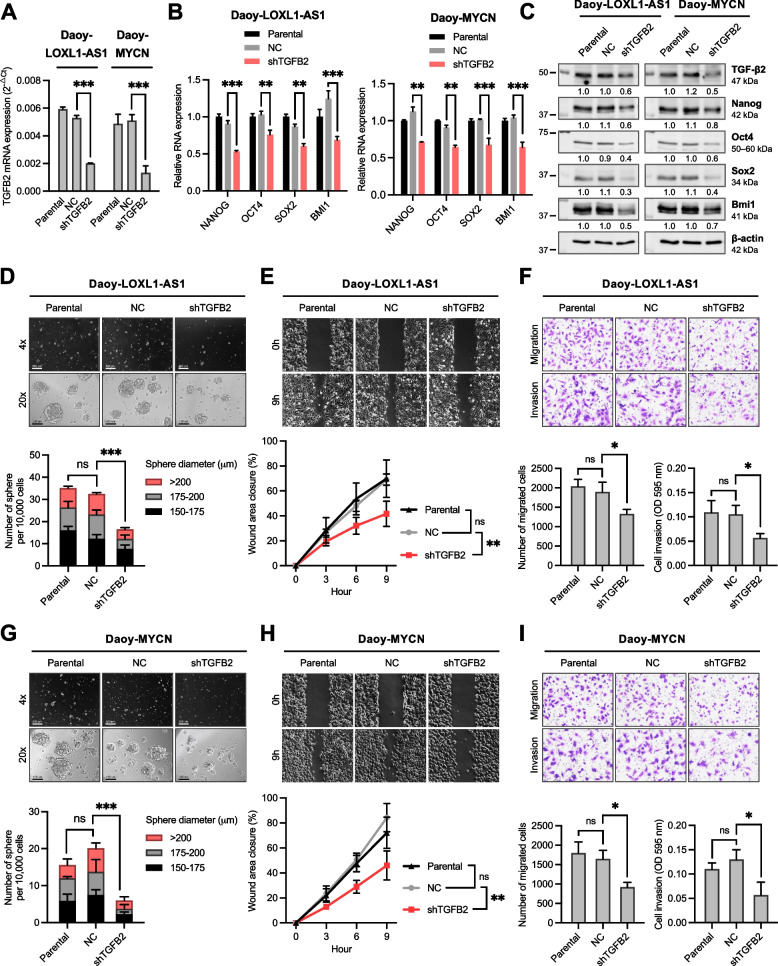
Effects of transforming growth factor (TGF)-β2 silencing on metastatic phenotypes of sonic-hedgehog medulloblastoma cells with high *LOXL1-AS1* expression. **A** RT-qPCR analysis of TGF-β2 expression in Daoy-LOXL1-AS1 and Daoy-MYCN cells. **B** RT-qPCR analysis of four stemness markers in Daoy-LOXL1-AS1 and Daoy-MYCN cells relative to the parental group. **C** Western blot analysis of four stemness markers in Daoy-LOXL1-AS1 and Daoy-MYCN cells. Quantification values below each band are relative to the parental group. **D**, **G** Sphere-formation assay of (D) Daoy-LOXL1-AS1 and (G) Daoy-MYCN cells, including microscopic images (left images), and the number and sizes of spheres of ≥150 μm in diameter (right bar chart). Scale bars, 500 μm in 4× and 100 μm in 20× magnification. **E**, **H** Wound-healing assay of (E) Daoy-LOXL1-AS1 and (H) Daoy-MYCN cells. **F**, **I** Transwell migration and invasion assays of (F) Daoy-LOXL1-AS1 and (I) Daoy-MYCN cells including quantification of cell migration (left bar chart) and invasion (right bar chart). Quantitative data are presented as mean ± SD of replicates from representative of three independent experiments. NC, negative control; ns, non-significant. * *p* < 0.05, ** *p* < 0.01, *** *p* < 0.001

Next, we defined the functional role of TGF-β2 by investigating whether it was sufficient to abrogate metastatic phenotypes promoted by *LOXL1-AS1* in SHH-MB cells. To this end, we used shRNA to silence endogenous TGF-β2 in Daoy-MYCN and Daoy-LOXL1-AS1 cells (Fig. 7A). The metastatic phenotypes of Daoy-LOXL1-AS1 cells were significantly impaired by TGF-β2 knockdown, reflected via decreases in expression of stemness markers (Fig. 7B-C), sphere formation (Fig. 7D), and cell migration and invasion (Fig. 7E-F). TGF-β2 knockdown in Daoy-MYCN cells also resulted in similar effects (Fig. 7B-C, G-I), which were previously observed under *LOXL1-AS1* silencing (Figs. 3-4). Taken together, TGF-β2 is regulated by *LOXL1-AS1* and mediates the prometastatic effects of *LOXL1-AS1* in SHH-MB cells.

## Discussion

Metastasis, either at diagnosis or at recurrence, is the adverse feature that contributes the most to mortality in MB [5]. In this study, we recapitulated metastasis in vivo using SHH-MB cells that expressed the MYCN malignant driver. An integrative screening approach was applied to identify the *LOXL1-AS1* lncRNA as a potential prometastatic factor. Recently, *LOXL1-AS1* has emerged as an oncogenic lncRNA in several cancer types [30, 31]. A study by Gao et al., to our knowledge the first and only report dedicated to *LOXL1-AS1* in MB, suggested its involvement in the aggressiveness of Group 3 MB [30]. Here, we focused on the SHH subgroup of MB in which *LOXL1-AS1* expression was highly prognostic. We further described in detail the contribution of *LOXL1-AS1* to critical aspects of the metastasis hallmark, including cancer stemness, and discovered TGF-β2 to be a mediator of these prometastatic functions in SHH-MB.

Apart from the high prognostic value of *MYCN* amplification status in SHH-MB, MYCN mRNA expression was elevated in WNT-MB and SHH-MB but its prognostic value remains inconclusive across all subgroups [12, 32, 33]. Our RNA-Seq validation confirmed the same findings, with the MYCN mRNA level being the highest but a suboptimal indicator of clinical outcomes in SHH-MB (Supplementary Fig. S2C-F). *LOXL1-AS1* expression, on the other hand, was not high in the SHH subgroup or any of its subtypes (Supplementary Figs. S2G-I), but was superior in correlating poor outcomes compared to MYCN. This highlights the pathological context-specific characters of lncRNAs like *LOXL1-AS1,* in contrast to MYCN which is a universal transcription factor with pleiotropic roles in both normal and disease processes [15].

In MB, metastasis occurs in a distinct way of leptomeningeal dissemination where cancer cells are seeded via cerebral spinal fluid to form multiple lesions across the central nervous system [34]. Stemness features of spreading MB cells, i.e., the cells’ ability to initiate and propagate to form a secondary population, is particularly relevant to this mode of metastasis. As suggested from our animal data, high *LOXL1-AS1* expression seemed to be an advantage for SHH-MB cells to establish metastatic tumors during leptomeningeal spread. This aligns with recent studies showing that primary and metastatic MB tissues have distinct genetic profiles [5] and clonal selection can give rise to divergent cell populations [35]. To this end, we further pointed out the previously unknown role of *LOXL1-AS1* in SHH-MB cell’s stemness. Among stemness markers mediated by *LOXL1-AS1*, Sox2 was highly expressed and was correlated with poor prognosis in the SHH subgroup [36, 37]. Sox2 expression defines both tumor-initiating and tumor-propagating MB cells capable of self-renewal and resistance to SMO inhibitors [38, 39]. Oct4 and Bmi1 are predictive of shorter survival times [40] and can maintain a stem-like cellular state to drive MB formation and metastasis [21, 41]. Bmi1 also regulates multiple genes associated with tumor-stromal interactions and cell adhesion in MB [42], whereas little is known about the role of Nanog in this tumor.

The TGF-β family is a well-established mediator of cancer stem cell potential and metastatic niches via either canonical Smad signaling or non-canonical pathways like PI3K/AKT/mTOR [43]. In brain malignancies, TGF-β1 and TGF-β2 are known as tumor growth factors [44], while a recent study revealed that TGF-β1 signaling from T cells can promote SHH-MB progression [45]. However, no previous study investigated the role of TGF-β2 in the SHH subgroup. Herein, we discovered TGF-β2 to be a *LOXL1-AS1*-dependent gene and confirmed its pro-metastatic functions in SHH-MB. While TGF-β2 and Sox2 were both transcriptionally mediated by *LOXL1-AS1*, these two genes were also strongly correlated in SHH-MB (Supplementary Fig. S5G). This indicates a crucial role of *LOXL1-AS1* in regulating a group of pro-stemness drivers to support SHH-MB metastasis.

The present study has several limitations. The exact mechanism of how MYCN regulates *LOXL1-AS1* remain to be determined, which can be via different mechanisms like transcriptional activation via direct promoter region binding [46] or indirectly via intermediate dependent factors [47]. The way *LOXL1-AS1,* TGF-β2, and other stemness regulators interact at the molecular level also warrant deeper investigation. The low number of cases in our MB cohort should also be noted when interpreting results from patient data, which was partially resolved by validation with a larger and non-overlapping MB cohort. Finally, our animal model performed in mice intracranially engrafted with SHH-MB cell lines can be further improved by using patient-derived primary cells collected at recurrence. Once overcoming the low establishment rate of only around 35% [48, 49], this type of model can more accurately recapitulate MB recurrence and metastasis in response to conventional therapies.

Findings about *LOXL1-AS1*’s specific expression, prometastatic function, and prognostic value in SHH-MB make it a promising candidate for translational studies. Since the analysis of disseminating tumor cells in the cerebrospinal fluid is emerging [50], biomarkers like *LOXL1-AS1* could be further evaluated for molecular stratifications to assist risk-adapted therapy. Furthermore, with recent advances in precise gene delivery and lncRNA breakdown in vivo, therapeutic models targeting *LOXL1-AS1* can be developed to reduce metastatic events in such high-risk cases of SHH-MB.

## Conclusions

In conclusion, the MYCN-associated lncRNA *LOXL1-AS1* contributes to SHH-MB metastasis by promoting TGF-β2-mediated cancer stem-like phenotypes. This study discovered a novel molecular target with both prognostic and therapeutic implications to minimize tumor recurrence, potentiating the de-escalation of cytotoxic regimens to improve clinical outcomes in children bearing this dismal type of brain tumor.

### Supplementary Information

**Additional file 1:.** This manuscript includes 1 additional data file: LOXL1-AS1_Supplementary-tables-figures.pdf

## Data Availability

Data generated and/or analyzed in the current study are available from the corresponding author upon reasonable request.

## Abbreviations

BLI: Bioluminescence imaging
DEG: Differentially expressed gene
EMT: Epithelial mesenchymal transformation
GSEA: Gene set enrichment analysis
lncRNA: Long non-coding RNA
MB: Medulloblastoma
RNA-Seq: RNA sequencing
SHH: Sonic-hedgehog
WNT: Wingless

## References

[1] Ostrom QT, Price M, Ryan K, Edelson J, Neff C, Cioffi G (2022). CBTRUS statistical report: pediatric brain tumor foundation childhood and adolescent primary brain and other central nervous system tumors diagnosed in the United States in 2014-2018. Neuro Oncol..

[2] Tulla M, Berthold F, Graf N, Rutkowski S, von Schweinitz D, Spix C (2015). Incidence, trends, and survival of children with embryonal tumors. Pediatrics..

[3] von Bueren AO, Kortmann RD, von Hoff K, Friedrich C, Mynarek M, Muller K (2016). Treatment of children and adolescents with metastatic Medulloblastoma and prognostic relevance of clinical and biologic parameters. J Clin Oncol.

[4] Mynarek M, von Hoff K, Pietsch T, Ottensmeier H, Warmuth-Metz M, Bison B (2020). Nonmetastatic Medulloblastoma of early childhood: results from the prospective clinical trial HIT-2000 and an extended validation cohort. J Clin Oncol.

[5] Ramaswamy V, Remke M, Bouffet E, Faria CC, Perreault S, Cho YJ (2013). Recurrence patterns across medulloblastoma subgroups: an integrated clinical and molecular analysis. Lancet Oncol.

[6] Hill RM, Kuijper S, Lindsey JC, Petrie K, Schwalbe EC, Barker K (2015). Combined MYC and P53 defects emerge at medulloblastoma relapse and define rapidly progressive, therapeutically targetable disease. Cancer Cell.

[7] Robinson GW, Rudneva VA, Buchhalter I, Billups CA, Waszak SM, Smith KS (2018). Risk-adapted therapy for young children with medulloblastoma (SJYC07): therapeutic and molecular outcomes from a multicentre, phase 2 trial. Lancet Oncol.

[8] Northcott PA, Buchhalter I, Morrissy AS, Hovestadt V, Weischenfeldt J, Ehrenberger T (2017). The whole-genome landscape of medulloblastoma subtypes. Nature..

[9] Cavalli FMG, Remke M, Rampasek L, Peacock J, Shih DJH, Luu B (2017). Intertumoral heterogeneity within Medulloblastoma subgroups. Cancer Cell.

[10] Lafay-Cousin L, Bouffet E, Strother D, Rudneva V, Hawkins C, Eberhart C (2020). Phase II study of nonmetastatic desmoplastic Medulloblastoma in children younger than 4 years of age: a report of the Children's oncology group (ACNS1221). J Clin Oncol.

[11] Tonn S, Korshunov A, Obrecht D, Sill M, Spohn M, von Hoff K (2023). Risk prediction in early childhood sonic hedgehog medulloblastoma treated with radiation-avoiding chemotherapy: evidence for more than 2 subgroups. Neuro-Oncology.

[12] Roussel MF, Robinson GW. Role of MYC in Medulloblastoma. Cold Spring Harb Perspect Med. 2013;3(11). 10.1101/cshperspect.a014308 PMC3808772 24186490

[13] Louis DN, Perry A, Wesseling P, Brat DJ, Cree IA, Figarella-Branger D (2021). The 2021 WHO classification of tumors of the central nervous system: a summary. Neuro-Oncology.

[14] Kool M, Jones DT, Jager N, Northcott PA, Pugh TJ, Hovestadt V (2014). Genome sequencing of SHH medulloblastoma predicts genotype-related response to smoothened inhibition. Cancer Cell.

[15] Shrestha S, Morcavallo A, Gorrini C, Chesler L (2021). Biological role of MYCN in Medulloblastoma: novel therapeutic opportunities and challenges ahead. Front Oncol.

[16] Ramaswamy V, Remke M, Bouffet E, Bailey S, Clifford SC, Doz F (2016). Risk stratification of childhood medulloblastoma in the molecular era: the current consensus. Acta Neuropathol.

[17] Schwalbe EC, Lindsey JC, Nakjang S, Crosier S, Smith AJ, Hicks D (2017). Novel molecular subgroups for clinical classification and outcome prediction in childhood medulloblastoma: a cohort study. Lancet Oncol.

[18] Li Y, Song Q, Day BW (2019). Phase I and phase II sonidegib and vismodegib clinical trials for the treatment of paediatric and adult MB patients: a systemic review and meta-analysis. Acta Neuropathol Commun.

[19] Swartling FJ, Savov V, Persson AI, Chen J, Hackett CS, Northcott PA (2012). Distinct neural stem cell populations give rise to disparate brain tumors in response to N-MYC. Cancer Cell.

[20] Huang M, Tailor J, Zhen Q, Gillmor AH, Miller ML, Weishaupt H (2019). Engineering genetic predisposition in human Neuroepithelial stem cells recapitulates Medulloblastoma tumorigenesis. Cell Stem Cell.

[21] Cancer M, Hutter S, Holmberg KO, Rosen G, Sundstrom A, Tailor J (2019). Humanized stem cell models of pediatric Medulloblastoma reveal an Oct4/mTOR Axis that promotes malignancy. Cell Stem Cell.

[22] Liu SJ, Dang HX, Lim DA, Feng FY, Maher CA (2021). Long noncoding RNAs in cancer metastasis. Nat Rev Cancer.

[23] Kesherwani V, Shukla M, Coulter DW, Sharp JG, Joshi SS, Chaturvedi NK (2020). Long non-coding RNA profiling of pediatric Medulloblastoma. BMC Med Genet.

[24] Skowron P, Farooq H, Cavalli FMG, Morrissy AS, Ly M, Hendrikse LD (2021). The transcriptional landscape of Shh medulloblastoma. Nat Commun.

[25] Wu KS, Sung SY, Huang MH, Lin YL, Chang CC, Fang CL, et al. Clinical and molecular features in medulloblastomas subtypes in children in a cohort in Taiwan. Cancers (Basel). 2022;14(21). 10.3390/cancers14215419 PMC9657873 36358838

[26] Hussein D, Punjaruk W, Storer LC, Shaw L, Othman R, Peet A (2011). Pediatric brain tumor cancer stem cells: cell cycle dynamics, DNA repair, and etoposide extrusion. Neuro-Oncology.

[27] Le TT, Hsieh CL, Lin IH, Chu CY, Do AD, Chen SH (2022). The ADAM9/UBN2/AKR1C3 axis promotes resistance to androgen-deprivation in prostate cancer. Am J Cancer Res.

[28] Ahmad Z, Jasnos L, Gil V, Howell L, Hallsworth A, Petrie K (2015). Molecular and in vivo characterization of cancer-propagating cells derived from MYCN-dependent medulloblastoma. PLoS One.

[29] Wang X, Venugopal C, Manoranjan B, McFarlane N, O'Farrell E, Nolte S (2012). Sonic hedgehog regulates Bmi1 in human medulloblastoma brain tumor-initiating cells. Oncogene..

[30] Gao R, Zhang R, Zhang C, Liang Y, Tang W (2018). LncRNA LOXL1-AS1 promotes the proliferation and metastasis of medulloblastoma by activating the PI3K/AKT pathway. Anal Cell Pathol (Amst)..

[31] Yu S, Gao X, Liu S, Sha X, Zhang S, Zhang X, et al. LOXL1-AS1 inhibits JAK2 ubiquitination and promotes cholangiocarcinoma progression through JAK2/STAT3 signaling. Cancer Gene Ther. 2024;31. 10.1038/s41417-024-00726-2 38267625

[32] Ryan SL, Schwalbe EC, Cole M, Lu Y, Lusher ME, Megahed H (2012). MYC family amplification and clinical risk-factors interact to predict an extremely poor prognosis in childhood medulloblastoma. Acta Neuropathol.

[33] da Silva LS, Mancano BM, de Paula FE, Dos Reis MB, de Almeida GC, Matsushita M (2020). Expression of GNAS, TP53, and PTEN improves the patient prognostication in sonic hedgehog (SHH) Medulloblastoma subgroup. J Mol Diagn.

[34] Fults DW, Taylor MD, Garzia L (2019). Leptomeningeal dissemination: a sinister pattern of medulloblastoma growth. J Neurosurg Pediatr.

[35] Morrissy AS, Garzia L, Shih DJ, Zuyderduyn S, Huang X, Skowron P (2016). Divergent clonal selection dominates medulloblastoma at recurrence. Nature..

[36] Ahlfeld J, Favaro R, Pagella P, Kretzschmar HA, Nicolis S, Schuller U (2013). Sox2 requirement in sonic hedgehog-associated medulloblastoma. Cancer Res.

[37] Vanner RJ, Remke M, Gallo M, Selvadurai HJ, Coutinho F, Lee L (2014). Quiescent sox2(+) cells drive hierarchical growth and relapse in sonic hedgehog subgroup medulloblastoma. Cancer Cell.

[38] Selvadurai HJ, Luis E, Desai K, Lan X, Vladoiu MC, Whitley O (2020). Medulloblastoma arises from the persistence of a rare and transient Sox2(+) granule neuron precursor. Cell Rep.

[39] Swiderska-Syn M, Mir-Pedrol J, Oles A, Schleuger O, Salvador AD, Greiner SM (2022). Noncanonical activation of GLI signaling in SOX2(+) cells drives medulloblastoma relapse. Sci Adv..

[40] Filipponi D, Pagnuzzi-Boncompagni M, Pages G. Inhibiting ALK2/ALK3 signaling to differentiate and chemo-sensitize medulloblastoma. Cancers (Basel). 2022;14(9). 10.3390/cancers14092095 PMC9102092 35565225

[41] Wiederschain D, Chen L, Johnson B, Bettano K, Jackson D, Taraszka J (2007). Contribution of polycomb homologues Bmi-1 and Mel-18 to medulloblastoma pathogenesis. Mol Cell Biol.

[42] Merve A, Dubuc AM, Zhang X, Remke M, Baxter PA, Li XN (2014). Polycomb group gene BMI1 controls invasion of medulloblastoma cells and inhibits BMP-regulated cell adhesion. Acta Neuropathol Commun..

[43] Derynck R, Turley SJ, Akhurst RJ (2021). TGFbeta biology in cancer progression and immunotherapy. Nat Rev Clin Oncol.

[44] Jennings MT, Kaariainen IT, Gold L, Maciunas RJ, Commers PA (1994). TGF beta 1 and TGF beta 2 are potential growth regulators for medulloblastomas, primitive neuroectodermal tumors, and ependymomas: evidence in support of an autocrine hypothesis. Hum Pathol.

[45] Gate D, Danielpour M, Rodriguez J, Kim GB, Levy R, Bannykh S (2014). T-cell TGF-beta signaling abrogation restricts medulloblastoma progression. Proc Natl Acad Sci USA.

[46] Wang PL, Teng L, Feng YC, Yue YM, Han MM, Yan Q (2022). The N-Myc-responsive lncRNA MILIP promotes DNA double-strand break repair through non-homologous end joining. Proc Natl Acad Sci USA.

[47] Tee AE, Ling D, Nelson C, Atmadibrata B, Dinger ME, Xu N (2014). The histone demethylase JMJD1A induces cell migration and invasion by up-regulating the expression of the long noncoding RNA MALAT1. Oncotarget..

[48] Grigore FN, Yang SJ, Chen CC, Koga T (2023). Pioneering models of pediatric brain tumors. Neoplasia..

[49] Roussel MF, Stripay JL (2020). Modeling pediatric medulloblastoma. Brain Pathol.

[50] Hagel C, Sloman V, Mynarek M, Petrasch K, Obrecht D, Kuhl J (2022). Refining M1 stage in medulloblastoma: criteria for cerebrospinal fluid cytology and implications for improved risk stratification from the HIT-2000 trial. Eur J Cancer.

