# Supplementary material for: LOXL1-AS1 contributes to metastasis in sonic-hedgehog medulloblastoma by promoting cancer stem-like phenotypes
Source: J Exp Clin Cancer Res. 2024 Apr 30;43:130. doi: 10.1186/s13046-024-03057-0 (PMC11059759; doi:10.1186/s13046-024-03057-0)
Supplement: Supplementary file 1 — Additional file 1:. This manuscript includes 1 additional data file: LOXL1-AS1_Supplementary-tables-figures.pdf [file 13046_2024_3057_MOESM1_ESM.pdf]

Additional information for

LOXL1-AS1 contributes to metastasis in sonic-hedgehog medulloblastoma by promoting cancer stem-like phenotypes

Anh Duy Do, Kuo-Sheng Wu, Shing-Shung Chu, Le Hien Giang, Yu-Ling Lin, Che-Chang Chang, Tai-Tong Wong, Chia-Ling Hsieh, and Shian-Ying Sung*

*Correspondence to: Chia-Ling Hsieh (chsieh2@dcb.org.tw), Shian-Ying Sung (ssung@tmu.edu.tw)

This file includes additional tables and figures:

Supplementary Table S1. shRNA targeting sequences used in the study

Supplementary Table S2. Gene-specific primer pairs used in qRT-PCR

Supplementary Figure S1

Supplementary Figure S2

Supplementary Figure S3

Supplementary Figure S4

Supplementary Figure S5

Supplementary Table S1. shRNA targeting sequences used in the study.

Target gene	Gene ID	shRNA name	Target sequence
LOXL1-AS1	*NR_040066.1*	shLOXL1-AS1 #1196	GGTGCATACAAGCACTCAA
LOXL1-AS1	*NR_040066.1*	shLOXL1-AS1 #2278	GGTCTTGGACTTTATTGTT
TGFB2	*NM_003238*	shTGFB2	CGGATTGAGCTATATCAGATT
Negative control	-	shNC	TTCTCCGAACGTGTCACGT

Supplementary Table S2. Gene-specific primer pairs used in qRT-PCR.

Gene name	Forward primer	Reverse primer
MYCN	CCACAAGGCCCTCAGTACC	TCTTCCTCTTCATCATCTTCATCA
LOXL1-AS1	AGTCCACAAATCCTAGGTGTA	CTCGTTTCCGATCCAGCCAGG
SOX2	TTGCTGCCTCTTTAAGACTAGGA	TAAGCCTGGGGCTCAAACT
OCT4	ATCGAGAACCGAGTGAGAGG	CACTCGGACCACATCCTTCT
NANOG	CTCCAACATCCTGAACCTCAGC	CGTCACACCATTGCTATTCTTCG
BMI1	AATCCCCACCTGATGTGTGT	GCTGGTCTCCAGGTAACGAA
TGFB2	ACACTCAGCACAGCAGGGTCCT	TTGGGACACGCAGCAAGGAGAAG
HOTAIR	CCAGAGAACGCTGGAAAAACCTG	GGAGATGATAAGAAGAGCAAGGAA
RAMP2-AS1	GAACTCAGGCCAGATTTACAAG	TTGGGTCCTACAGCAACCAT
LINC01606	GCTGGACATTTCTCCCTTCA	GAGTCCTCTCGCTTCCTCCT
TRHDE-AS1	GGGTGTAGAGAGGGAAGTTAGG	TTCTCTCCAGCTGCAGGGTGTA
CLMN	AGGCTTTCAGCATCGCACAGGA	CTAGAAACTGTGCCACGTAAGTC
KCNQ3	CGTCTGATTGCCGCCACCTTTT	TTCTGACGGTGTTGCTCCTGCA
USP2	GAGATACGCACCGCGCTTTGTT	GGTTGGACTTAGGTCTCAGTGTC
LOXL1	ACAGCACCTGTGACTTCGGCAA	CGGTTATGTCGATCCACTGGCA
SAMD11	GAGCATCAGAGCCGCTGTGAAT	AAAGCAGTCGCTGCTGATACGG
HSPCB	AGCCTACGTTGCTCACTATTACG	GAAAGGCAAAAGTCTCCACCT

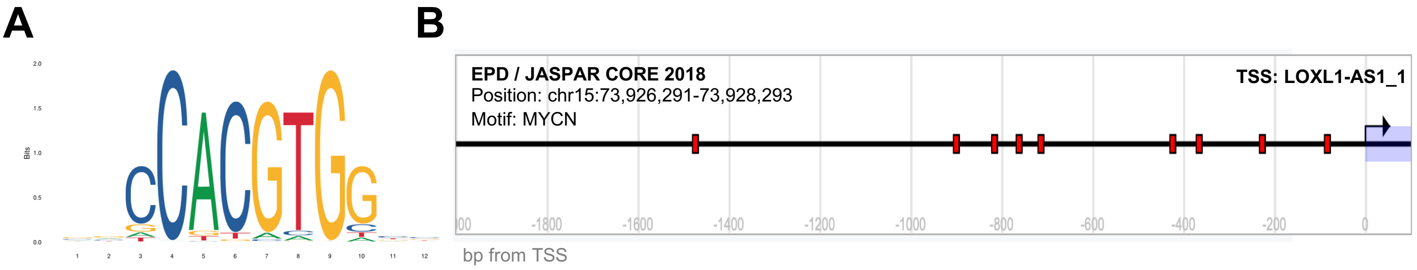

Supplementary Figure S1. A genomic region of 2-kilo base pairs upstream of the transcription starting site (TSS) of *LOXL1-AS1* transcript 1 (NR_040066) was used as input for screening of transcription factor binding sites using EPD (Eukaryotic Promoter Database, https://epd.expasy.org/) and JASPAR 2018 (https://jaspar.genereg.net) databases. **(A)** Illustration of MYCN binding motif. **(B)** Detection of MYCN binding motif within the genomic region of interest.

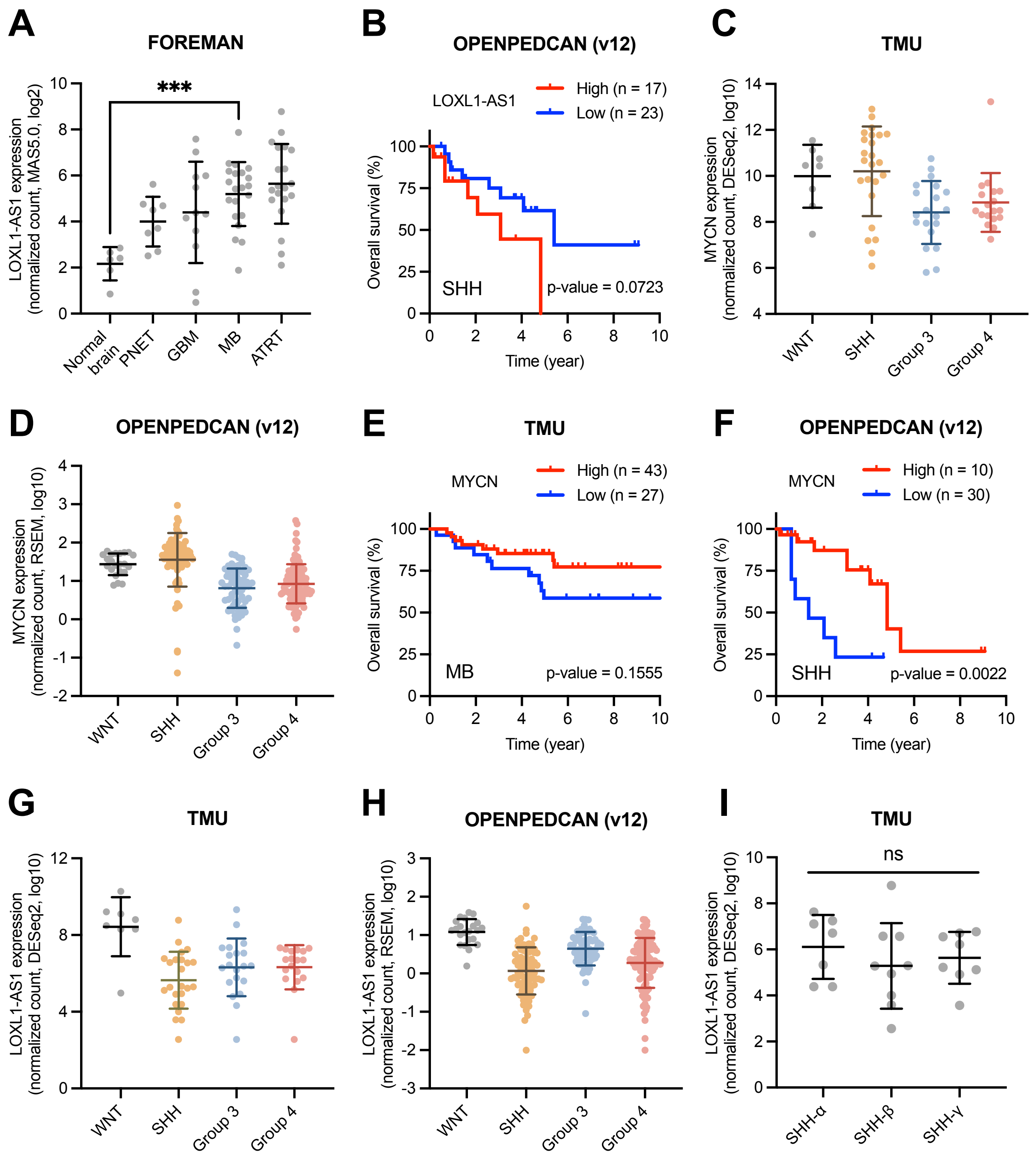

Supplementary Figure S2. (A) *LOXL1-AS1* expression in the normal brain and different types of brain tumor in a public dataset (Foreman, R2 platform). PNET, primitive neuroectodermal tumor; GBM, glioblastoma; MB, medulloblastoma; ATRT, atypical teratoid/rhabdoid tumor. **(B)** Survival analyses of *LOXL1-AS1* expression in SHH-MB in the OpenPedCan cohort. **(C-D)** MYCN expression level across four molecular subgroups in the TMU and OpenPedCan cohorts. **(E-F)** Survival analyses of MYCN expression in the TMU and OpenPedCan cohorts. **(G-I)** *LOXL1-AS1* expression in the TMU and OpenPedCan cohorts, including (G-H) expression level across four molecular subgroups of MB and (I) expression level across three molecular subtypes of SHH-MB. ns, non-significant; *** p <0.001

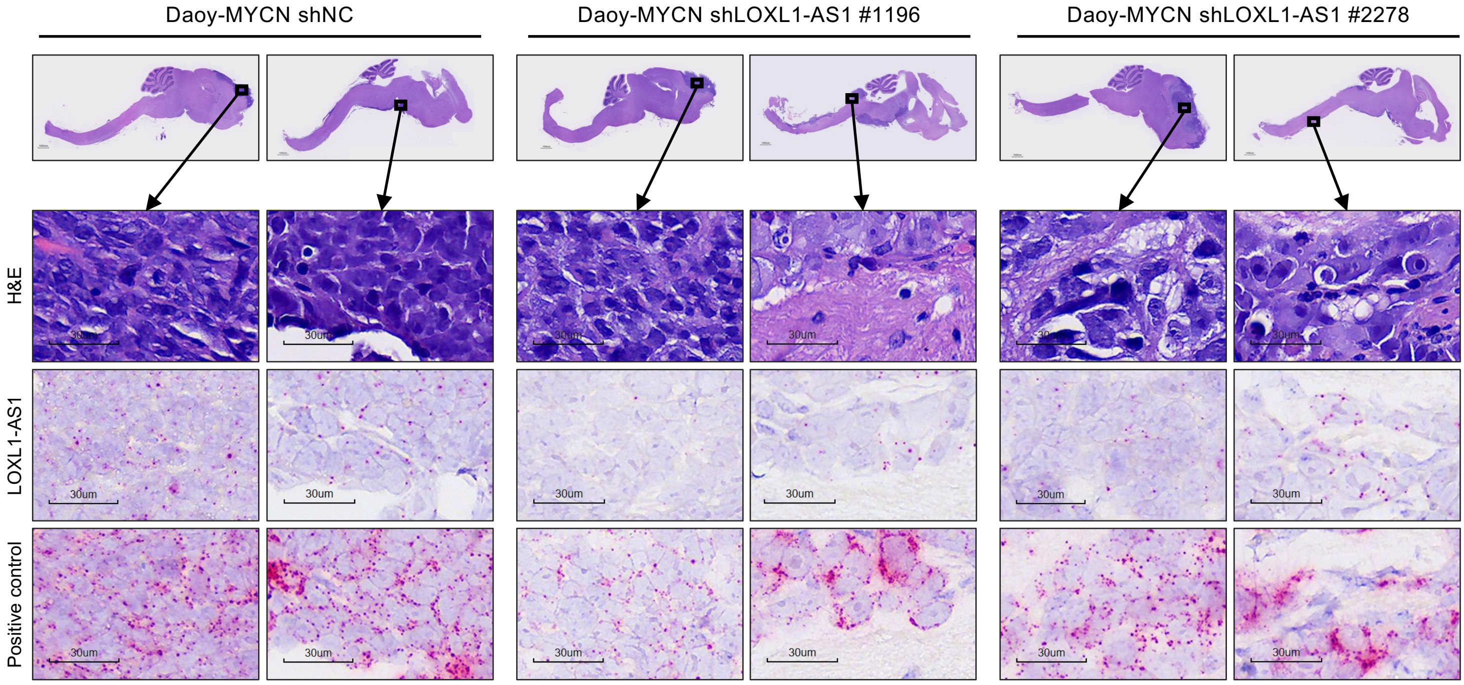

Supplementary Figure S3. (A) H&E staining and *in situ* hybridization of *LOXL1-AS1* and positive control RNA in the brain and spinal cord tissue of representative mice from each group. Scale bars 30 μm.

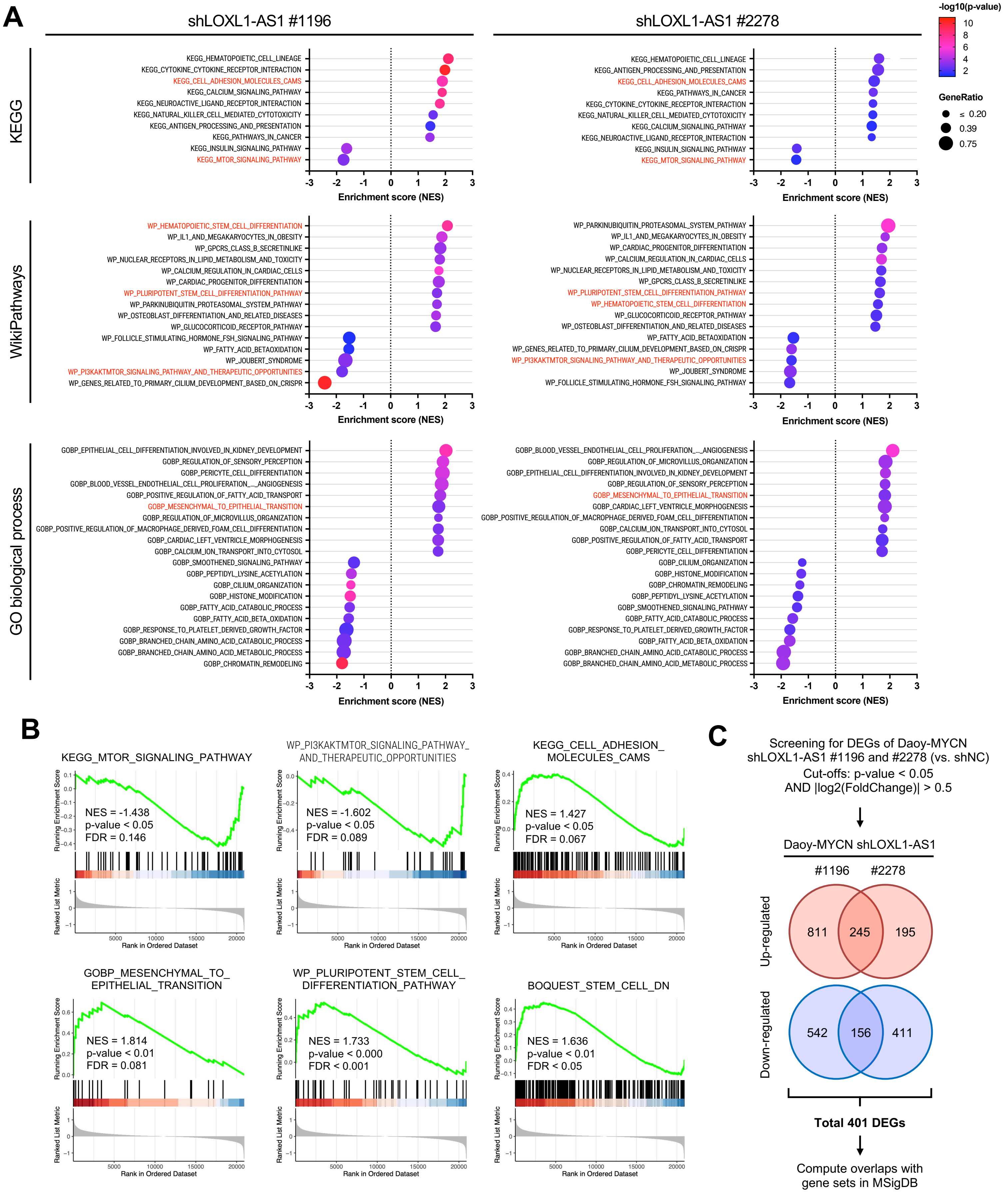

Supplementary Figure S4. (Legend next page)

Supplementary Figure S4. (A) Gene set enrichment analysis (GSEA) of Daoy-MYCN-shLOXL1-AS1 #1196 (left) and #2278 (right) in terms of normalized enrichment score (NES), p-value and gene ratio. Three gene set collections of KEGG, WikiPathways, and GO biological process are presented with top 10 up-regulated and top 10 down-regulated gene sets in each collection. Gene sets of interest are highlighted in red. **(B)** GSEA plots of Daoy-MYCN-shLOXL1-AS1 #2278 versus shNC samples in six gene sets of interest. **(C)** Schematic outline describing the workflow of selecting significant DEGs from two shLOXL1-AS1 knockdown samples. A total of 401 DEGs were input for computing overlaps with other gene sets using the Molecular Signature Database (MSigDB, www.gsea-msigdb.org) in Figure 6C.

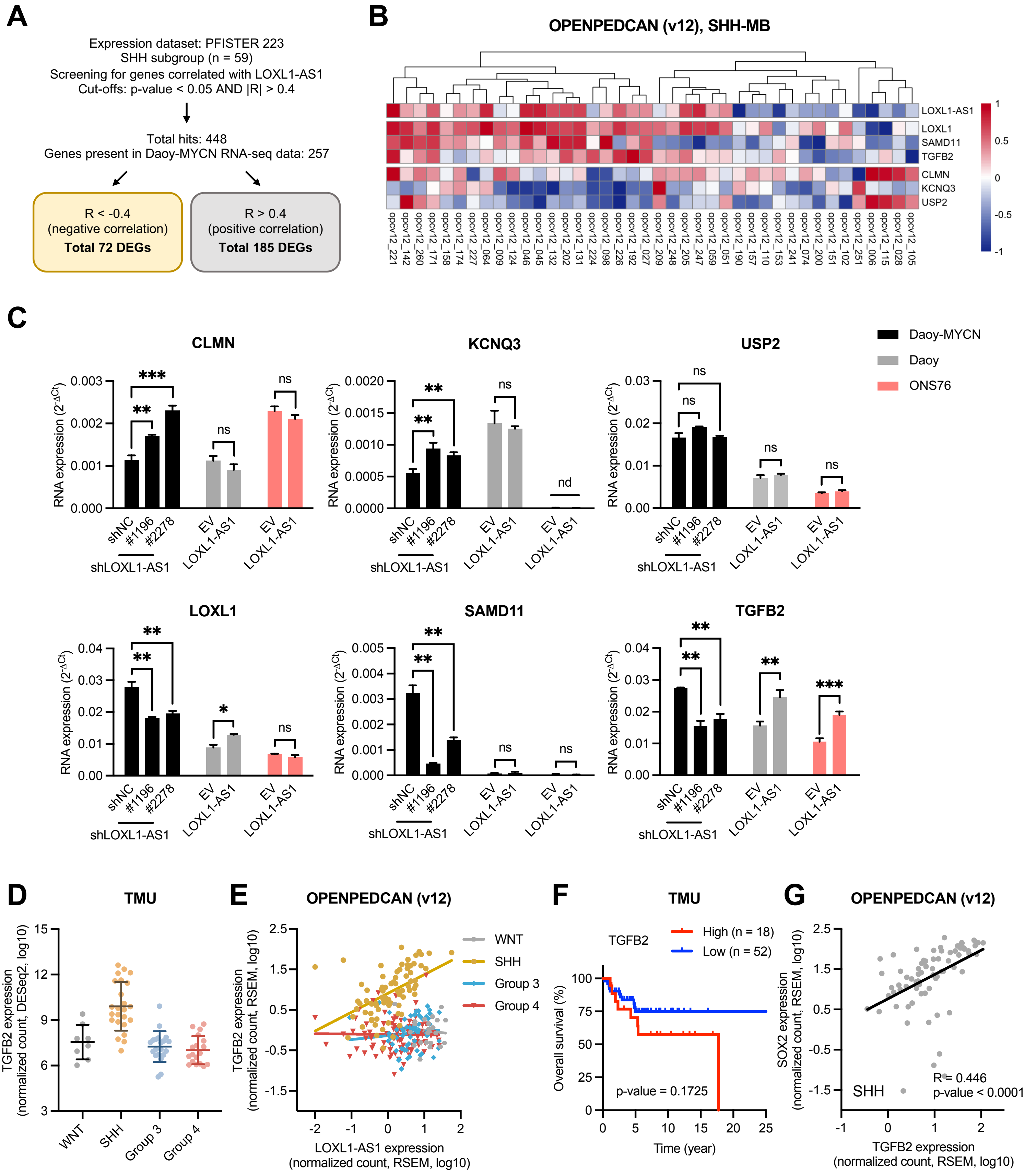

Supplementary Figure S5. (Legend next page)

Supplementary Figure S5. (A) Schematic outline describing the workflow of screening for genes correlated with *LOXL1-AS1* in a public dataset (Pfister-223, SHH-MB, n=59). **(B)** *LOXL1-AS1* expression of 6 candidate genes in OpenPedCan cohort (SHH-MB, n=40). **(C)** qRT-PCR validation of 6 candidate genes in SHH-MB cell lines. **(D)** *TGFB2* expression across four molecular subgroups in TMU cohort. **(E)** Correlation between *TGFB2* and *LOXL1-AS1* across four molecular subgroups in OpenPedCan cohort. **(F)** Survival analysis of *TGFB2* in TMU cohort. **(G)** Correlation between *TGFB2* and *SOX2* in SHH-MB in OpenPedCan cohort.
